# Lkb1 aggravates diffuse large B-cell lymphoma by promoting the function of Treg cells and immune escape

**DOI:** 10.1186/s12967-022-03588-0

**Published:** 2022-08-19

**Authors:** Xiuhua Su, Tao Sun, Meng Li, Yuan Xia, Mingying Li, Dongmei Wang, Fei Lu, Jingjing Ye, Chunyan Ji

**Affiliations:** 1 Department of Hematology, Qilu Hospital, Cheeloo College of Medicine, Shandong University, Jinan, 250012 People’s Republic of China; 2 School of Medicine, Cheeloo College of Medicine, Shandong University, Jinan, 250012 China; 3 Shandong Key Laboratory of Immunohematology, Qilu Hospital, Cheeloo College of Medicine, Shandong University, Jinan, 250012 People’s Republic of China

**Keywords:** Diffuse large B-cell lymphoma, Regulatory T cells, Lkb1, Immunosuppression, Tumor immune microenvironment

## Abstract

**Background:**

Regulatory T cells (Tregs) induce immune responses and may contribute to immune escape in tumors. Accumulation of Tregs in tumors represents a critical barrier to anti-tumor immunity and immunotherapy. However, conflicting results describing the role of Tregs in lymphoma warrant further investigation. The precise features and mechanisms underlying the alteration in Tregs in diffuse large B-cell lymphoma (DLBCL) are not well understood yet. In this study, we analyzed the mechanism underlying the observed alterations in Tregs in DLBCL and examined the effect of Lkb1 expression on the immunosuppressive function of human Tregs.

**Methods:**

Flow cytometry and immunofluorescence were used to analyze the proportion of Tregs and effector Tregs in the peripheral blood and lymph nodes of patients with DLBCL and control group. In vitro culture assays were used to analyze the immunosuppressive function of Tregs in the two groups. Transcriptome sequencing was performed to analyze the differentially expressed genes in the two groups, and the transcription level and protein expression of Lkb1 in the two groups were detected using RT-PCR and WES microprotein technology. Lentiviral vectors were constructed to explore the functional changes of Tregs with stable upregulation and downregulation of Lkb1. Finally, a humanized murine lymphoma model was established to study the function of Lkb1 in Tregs in the pathogenesis of DLBCL.

**Results:**

The number of Tregs was found to be dramatically increased in peripheral blood and tumor tissue in DLBCL patients compared with that in healthy controls, and decreased after treatment. Tregs from DLBCL patients exhibited multiple enhanced functions, including increased inhibition of CD8^+^cytotoxic T cells (CTL) against tumor cells, enhanced suppression of CD8^+^CTL secretion of granular enzyme, and suppression of CD8^+^CTL degranulation. Lkb1 was found to be upregulated in Tregs of DLBCL patients. Furthermore, Lkb1 contributes to Treg immunosuppressive function in DLBCL by regulating the mevalonate pathway. Finally, deletion of Lkb1 in Tregs suppressed tumor growth and promoted anti-tumor immunity in a DLBCL murine model.

**Conclusions:**

These findings confirmed that Lkb1-regulated Tregs are critical for immune escape in DLBCL, which emphasizes that Lkb1 is a potential target for the immunotherapy of DLBCL.

**Supplementary Information:**

The online version contains supplementary material available at 10.1186/s12967-022-03588-0.

## Background

Diffuse large B-cell lymphoma (DLBCL), characterized by lymphadenopathy, extranodal disease, or both, is the most common subtype of non-Hodgkin lymphoma and represents approximately 30–40% of newly diagnosed cases [1]. DLBCL is a highly aggressive and heterogeneous lymphoma. Chemotherapy with rituximab is considered the first-line therapy for DLBCL, with a total effective rate of 60–70%. However, approximately one third of DLBCL patients develop relapsed/refractory diseases after chemotherapy [2]. The tumor microenvironment (TME) has been reported to be important to lymphoma and its dysfunction contributes to the pathogenesis and progression of the disease [3–5]. Specifically, an immunosuppressive TME may allow tumor cells to uncontrollably proliferate and remain undetected by the immune system. Regulatory T cells (Tregs) are vital for the modulation of the immune response, suppression of the activation of other immune cells, and maintaining immune system homeostasis [6]. Treg cells are with heterogeneous phenotypes and functions that can originate in the thymus (natural Tregs) or in the peripheral tissues (induced Tregs) [7]. According to the expression level of key markers, Tregs can be subdivided into three subpopulations: CD45RA^+^Foxp3^low^CD25^low^ (naïve Tregs), CD45RA^−^Foxp3^high^CD25^high^ (effector Tregs), and CD45RA^−^Foxp3^low^CD25 ^low^ (non-Tregs) [8].

Treg abundance has been shown to be correlated with a worse prognosis in many types of cancers, such as cervical cancer, breast cancer, and ovarian cancer [9–11]. However, the role of Tregs in hematologic or lymphoid malignancies such as DLBCL has been controversial. While some studies have verified that the increasing tissue infiltration of Tregs is associated with better clinical prognosis [12, 13], recent studies have shown that the increased proportion of Tregs in the peripheral blood mononuclear cells (PBMC) and TME of DLBCL patients is negatively correlated with tumor progression and prognosis [14–17]. This discrepancy may be attributed to the complexity of Treg interactions with other immune cell populations that promote or impede lymphoma growth, along with the diversity of Tregs and the different experimental approaches used for Treg detection. In addition, the mechanism underlying the alteration in the proportion and function of Tregs in patients with DLBCL has not been fully elucidated yet.

Liver kinase B1 (Lkb1), encoded by the STK11 gene, is a serine/threonine kinase and a bioenergetic sensor that controls cell metabolism and growth [18]. Additionally, Lkb1 phosphorylates AMPK, which also coordinates cell growth and metabolism [19]. In Tregs, Lkb1 stabilizes Foxp3 expression by preventing STAT4-mediated methylation of conserved non-coding sequences in the Foxp3 locus in an inflammatory environment [20]. Meanwhile, Foxp3 regulates the metabolic reprogramming of Tregs necessary to acquire functional activation that suppresses CD4^+^T cells [21, 22]. However, the contribution of Lkb1 regulation to tumor progression, as well as to the regulation of the TME, remains unclear.

In this study, we analyzed the abundance and function of Tregs in DLBCL. We further investigated the alteration of highly immunosuppressive effector Tregs and the mechanism underlying the observed alterations in Tregs. We examined the effect of Lkb1 expression on the immunosuppressive function of human Tregs as well as the activity of the mevalonate pathway. Our results might reveal an important mechanism of lymphoma immune escape mediated by Tregs and suggest that Lkb1-related pathways may be potential therapeutic targets for DLBCL.

## Materials and methods

### Patient characteristics

The study was approved by the Medical Ethical Committee of Qilu Hospital of the Shangdong University and was performed according to the Declaration of Helsinki. There were 45 patients with DLBCL newly diagnosed in the study, and 45 healthy donors were enrolled as the control group. All patients signed an informed consent form for obtaining samples by donation of PBMC. DLBCL was diagnosed by surgical removal of the bulking lymph node and pathological examination. Staging was verified according to the Ann Arbor system [23]. PBMC from healthy donors and reactive lymph nodes were used as controls. Patient characteristics are listed in Table 1. All the patients received the treatment with the standard immunochemotherapy R‐CHOP (rituximab, cyclophosphamide, doxorubicin, vincristine, and prednisone). All initial samples were obtained before any anti-lymphoma treatment was administered. There were sixteen samples collected again from DLBCL patients after three-six cycles of therapy, which including the complete remission (n = 11) and no remission (n = 5) patients after treatment.

**Table 1:** Clinical characteristics of DLBCL patients

Clinical characteristics	DLBCL patients	Healthy donors
Median age (range), years	56 (28–84)	55(28–80)
Male, n	25	25
Female, n	20	20
Lactate dehydrogenase level		
Normal	31	Null
Elevated	14	Null
Ann Arbor Stage		
I–II	13	Null
III–IV	32	Null
Cell of origin		
GCB	26	Null
Non-GCB	19	Null
IPI		
0–2	26	Null
3–5	19	Null
Location		
Nodal	16	Null
Extranodal	29	Null
B symptom		
Yes	13	Null
No	32	Null

*DLBCL* diffuse large B-cell lymphoma, *GCB* germinal center B-celllike, *IPI* international prognostic index

### Cell lines and mice

The human DLBCL cell line SU-DHL4 and the murine B-lymphoma cell line A20 were obtained from the American Type Culture Collection. SU-DHL4 cells were cultured in RPMI 1640 medium (Invitrogen Life Technologies, Waltham, MA, USA) supplemented with 10% fetal bovine serum (FBS, Biological Industries, Israel) and 1% penicillin/streptomycin (both from Invitrogen Life Technologies). A20 cells were cultured in RPMI 1640 medium with 10% FBS, 0.1 mM β-mercaptoethanol, and 1% penicillin/streptomycin. The cells were grown in a suspension culture at 37 °C in 5% CO_2_. All cell lines were recently authenticated based on short tandem repeat profiling and tested to be free of mycoplasma contamination.

Female BALB/c and NOD.Cg-Prkdcscid Il2rgtm1Wjl/SzJ (NSG) mice (aged 5–6 weeks) were purchased from The Jackson Laboratory and were housed under specific pathogen-free conditions at the Laboratory Animal Facility at Shandong University.

### In vivo tumor transplantation

To establish tumors, 5 × 10^6^ live A20 lymphoma cells were resuspended in 100 μL of phosphate-buffered saline and subcutaneously injected into the right back flank of BALB/c mice. Tumor size and body weight were measured at least twice a week. Animals bearing tumors were euthanized when tumors reached a size of 15 mm in diameter or earlier.

NSG mice were injected via the tail vein with 2 × 10^6^ SU-DHL4 cells. Then freshly prepared CD8^+^CTL and Tregs subjected to various treatments were intravenously injected into each mouse once a week for 3 weeks. Mice were monitored for clinical signs (ruffled fur, decreased activity, and tumor development) and survival. They were euthanized when they showed signs of distress and could not reach food or water. There were between five and six mice in each group.

### Flow cytometry

Fresh tumor tissue and reactive lymph nodes were ground to obtain a single-cell suspension and were treated with red blood cell lysis buffer (eBioscience, San Diego, CA, USA). PBMC were isolated using Ficoll–Hypaque centrifugation. The spleens, livers, and tumors of mice were also processed into single-cell suspensions. Cells were washed with phosphate-buffered saline containing 2% FBS and were incubated with CD3 (Biolegend, 300318), CD4 (Biolegend, 357410), CD8 (Biolegend, 344703), CD25 (Biolegend, 302610), CD45RA (Biolegend, 304148), CD127 (Biolegend, 351304) antibodies, CD107a (Biolegend, 328607), CD62L (Biolegend, 104411), and anti-mouse CD25 (Biolegend, 102007), anti-mouse CD4 (Biolegend, 100509). As CD127 expression inversely correlates with Foxp3 levels in CD25^+^Treg cells, the gate of CD127^lo^ is currently used to define Tregs using flow cytometry [24]. For intracellular Foxp3 (Biolegend, 320007), ki67 (Biolegend, 151211) and granzyme B (GzmB) (Biolegend, 372207) staining, cells were first stained with surface antibodies, and then fixed, permeabilized, and stained with anti-Foxp3, anti-ki67 and anti-GzmB for 50 min in the dark. Flow cytometry data were acquired on a BD FACSMelody device and analyzed using FlowJo (FlowJo LLC, Ashland, OR, USA). For in vitro functional assays, specific cell populations were isolated using cell sorting with a BD FACSMelody device, and the sorted populations were > 98% pure unless otherwise specified.

### RNA isolation and RT-PCR

Total RNA was extracted using the RNeasy Mini Kit (Qiagen) from Tregs purified from patients and healthy donors according to the manufacturer’s instructions. The extracted RNA was measured by calculating the absorption ratio of 260/280 using a Nanodrop spectrophotometer (Thermo Fisher, USA). Gene expression was measured using the GeneAmp 7500 Sequence Detection System (ABI). GAPDH was used as the reference gene for each sample. After the reactions were complete, the cycle threshold (CT) values were determined, and the mean CT of independent triplicates was determined.

### RNA sequencing and bioinformatic analysis

Total RNA was extracted with the RNeasy Mini Kit (Qiagen) from Tregs that had been purified by fluorescent-activated cell sorting from patients with DLBCL and healthy donors. RNA was then reverse transcribed and amplified, and sequencing libraries were generated using the NEBNext® Ultra™ RNA Library Prep Kit for Illumina® (NEB, Ipswich, MA, USA). Ultimately, after quantifying and qualifying the RNA-seq libraries, the sequencing is detected by Illumina Hiseq 4000. DEGs between each of the two groups were presented. To discover the potential underlying biological procedures and pathways in DLBCL and control group, we conducted GO and KEGG pathway analysis.

### Histopathology and immunofluorescence

Histopathological examinations were performed as previously reported. Tissues fixed in 4% paraformaldehyde were sectioned after paraffin embedding. Many sections were prepared and stained with hematoxylin and eosin for morphological observations.

Sections were deparaffinized, and antigen retrieval was performed for 15 min. Tissue sections were then incubated with anti-Foxp3 (Abcam, Cambridge, UK) overnight at 4 °C. After washing, the sections were incubated with secondary antibody (Life Technologies, Carlsbad, CA, USA) for 1 h. The sections were visualized under a light microscope.

### Western blotting

The sample protein was extracted from sorted Tregs after the cells were lysed and centrifuged at 12,000 × *g* for 15 min at 4 °C. The relative abundance of a protein level was quantified using an automatic Simple Western system (Santa Clara, CA, USA), as previously described [25]. For analysis of Lkb1 levels, cell lysates were diluted to a final concentration of 0.5 mg/mL and loaded in a microplate (Protein Simple #043-165), along with a monoclonal mouse anti-Lkb1 antibody (1:50; Invitrogen 206R24). Western blotting was conducted according to the instructions for the Wes system, which differ from those of the traditional method. Protein levels were analyzed using the Compass software (Protein Simple).

### T-cell proliferation assay

A total of 5 × 10^4^/well CD8^+^ CTL labeled with Tag-it VioletTM proliferation and cell tracking dye kit (Biolegend) were seeded into each well of a 96-well U-bottom plate and stimulated with human CD3/CD28 T cell activator (Stem cell, 10971) and recombinant human (rh) interleukin (IL)-2 (100 ng/mL, Peprotech) in RPMI-1640 medium (10% FBS). Freshly isolated Tregs were mixed with responders at a ratio of 1:2. Cells were harvested after 72 h, washed, and stained with antibodies for 30 min at 4 °C. The Tag-it Violet intensity was analyzed using flow cytometry.

### Cytotoxicity assay

Target cells (DLBCL cell lines) were labeled with 5,6-carboxyfluorescein-diacetatesuccinimidyl-ester ester (CFSE, Invitrogen Life Technologies) and cultured in 48-well plates along with different amounts of effector cells in RPMI-1640. Effector cells including CD8^+^CTL with or without Tregs from patients or healthy donors were incubated in the presence of human CD3/CD28 T cell activator and rhIL2.

When we co-cultured CD8^+^ CTLs with Tregs from healthy donors in which Lkb1 had been silenced or overexpressed, the target cells were labeled with CellTraceTM Far Red (Invitrogen Life Technologies). To determine cell viability, cells were stained with propidium iodide (PI) (BD Biosciences, San Jose, CA) and analyzed using flow cytometry. CD8^+^CTLs were analyzed for granzyme B expression, which is an indication of the cytotoxic killing effect of activated T cells.

### Degranulation assay

Degranulation was assessed by determining CD107a mobilization on CD8^+^CTL as previously described [26]. Briefly, CD8^+^CTLs with or without Tregs from patients or healthy donors were incubated with target cells (DLBCL cell lines) in 48-well plates in complete RPMI-1640 culture media at 37 °C for 4–5 h in the presence of CD107a. The cells were then stained for CD8 and analyzed using flow cytometry.

### Lentivirus production and Treg transduction

Lentiviral vectors pCDH-CMV-MCS-EF1-copGFP containing non-targeting control short hairpins and short hairpins targeting human *STK11* were produced in 293 T cells. To overexpress the human *STK11*, the canonical CDS at each gene locus was obtained from NCBI GenBank. After necessary sequence optimization for gene cloning, DNA sequences were synthesized and sub-cloned into the lentivirus vector. Lentiviral constructs repressing or expressing Lkb1 were purchased from GenePharma (Shanghai, China). Isolated Tregs were cultured in plates with human CD3/CD28 T cell activator and transduced in lentivirus-containing media by centrifugation for 1 h at 1000 × *g* in the presence of polybrene and rhIL-2. Tregs were transduced with lentivirus encoding human shRNA targeting human *STK11*. Viral supernatants were removed on day 3, and the infected Tregs were sorted for further analysis.

### Statistical analysis

Flow cytometry data were analyzed using FlowJo 10 (BD Biosciences). Kaplan–Meier plots were used to analyze survival. Survival curves were compared using log-rank tests. Statistical analysis was performed using Prism 9 software (GraphPad) by applying an unpaired Student’s t-test or one-way or two-way ANOVA with Bonferroni’s multiple comparison tests. Significance was set at P < 0.05.

## Results

### Increased Treg abundance within the tumor tissue and PBMC of patients with DLBCL

**Fig. 1:**
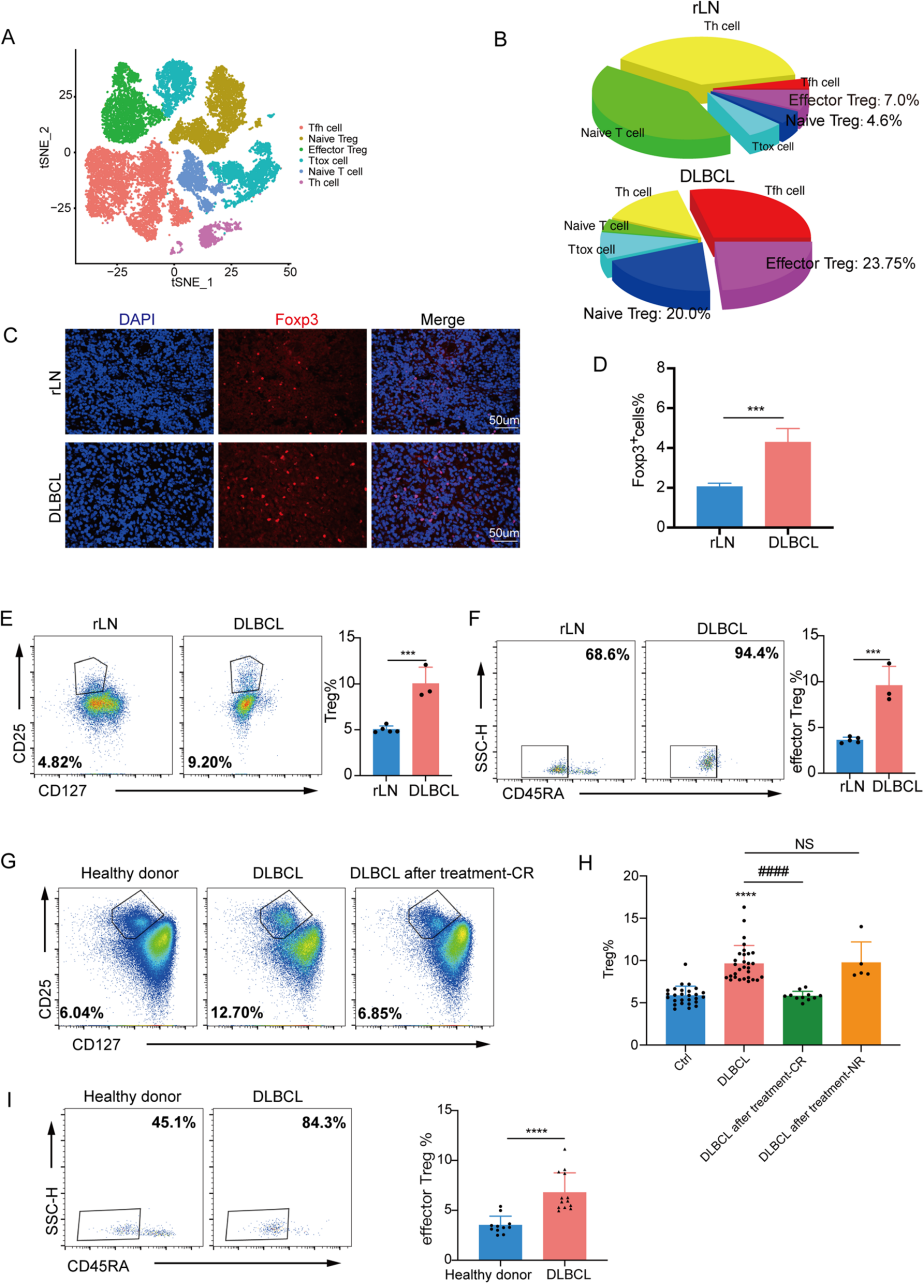
Increased Treg abundance within the tumor tissue and PBMC of patients with DLBCL. **A** T cells from DLBCL (n = 4) and reactive lymph nodes (n = 3) biologically independent samples were combined and jointly visualized using t-SNE. The cells were coloured with respect to their cluster. **B** Proportion of Treg cells and Treg subpopulations identified in samples from the two group based on scRNA-seq in pie charts. **C** Immunofluorescence staining of Treg cells from different groups for Foxp3 (red) and DAPI (blue). **D** The quantification of immunofluorescence staining of Foxp3^+^ cells in lymphatic tissues from DLBCL patients and reactive lymph node. Frequencies of CD4^+^ CD25^high^ CD127^low/neg^ Tregs (**E**) and CD45RA^−^ effector Tregs (**F**) in infiltrating lymphatic tissues of DLBCL patients (n = 3) and lymph node tissue from control group (n = 5). **G** Representative flow cytometric analysis of Tregs frequencies in PBMC sample from healthy donors (n = 27), DLBCL patients (n = 30) and the patients after treatment (n = 16). Within the CD4^+^ T cell gate, Tregs were identified as CD25^high^ CD127^low/neg^. **H** Frequencies of Tregs in PBMC from healthy donors (n = 27), DLBCL patients (n = 30) and the complete remission (n = 11) and no remission (n = 5) patients after treatment. **I** Representative flow cytometric analysis of CD45RA^−^ effector Tregs in each Treg subset frequencies in PBMC sample from healthy donors and DLBCL patients. The percentages of effector Tregs within the CD4^+^ T cell gate in PBMC from healthy donors (n = 11) and DLBCL patients (n = 13) were shown. *P < 0.05; **P < 0.01, ***P < 0.001. Asterisk indicates significant difference from control group as assessed. Pound sign indicates significant difference from different Treg group. In **H**, p values were determined by one-way ANOVA; In **D**–**F** and **I**, p values were determined by two-sided unpaired t-test; data are presented as mean values ± SEM

In the present study, using two published scRNA-seq databases [27, 28], we analyzed the lymphoma microenvironment, especially T cells. All T cells from either reactive or DLBCL lymph nodes were distributed to different T-cell subpopulations. Among them, we specifically focused on Tregs and Treg subsets (Fig. 1A). The abundance of Tregs was significantly increased in DLBCL tissues compared to that of the lymph node tissue of the control group, as well as the proportion of effector Tregs in pie charts (Fig. 1B). According to previously defined markers of naïve and effector Tregs [29], such as TCF7, CCR7, HLA-DR, and Foxp3, we subdivided Tregs into naïve and effector Tregs. The top signature genes of each cluster perfectly matched naïve status with CCR7^hi^TCF7^hi^HLA-DR^low^Foxp3^low^ profile were termed naïve subsets. The others with CCR7^low^TCF7^low^HLA-DR^hi^Foxp3^hi^ profile were effector subsets (Additional file 2: Fig. S2A). We repeated analysis in additional datasets from other diseases, such as acute myeloid leukemia (AML) [30] and T cell lymphoma [31]. Interestingly, data uncovered that the abundance of Tregs was significantly increased in AML and T cell lymphoma tissues compared to that of the control group, as well as the proportion of effector Tregs (Additional file 2: Fig. S2B-E). To further validate the findings in DLBCL in a larger cohort, we analysed the variable frequency of Tregs form 45 pairs of DLBCL-control human samples by immunofluorescence staining and flow cytometry. We assessed immunofluorescence staining of Foxp3, a marker of Treg cells, from different groups for Foxp3 (red) and DAPI (blue) in lymphoid tissue. The results indicated that increased expression of Foxp3 in DLBCL patients compared to that in the control group (Fig. 1C, D). As shown in a representative case in Fig. 1E, F and Additional file 1: Fig. S1B, the proportion of CD4^+^ CD25^high^ CD127^low/neg^ Tregs was significantly increased in lymphoma tissues from DLBCL and presented low CD45RA expression, indicating that more cells were the CD4^+^ CD25^high^ CD127^low/neg^ CD45RA^−^ effector subsets of Tregs. Co-expression of CD25^high^ and CD127^low/neg^ was used to identify and isolate Tregs [24]. We also demonstrated high Foxp3 expression in this population (Additional file 1: Fig. S1A), and we used this strategy to analyze the proportion of Treg cells because Tregs needed to be sorted for RNA sequencing and functional experiments. To investigate the role of Tregs in the peripheral immune system in DLBCL, we measured the abundance of Tregs in PBMC samples. The proportion of Tregs of the total CD4^+^ T cells detected in the PBMC of DLBCL patients was higher than that detected in healthy donors (P < 0.0001) (Fig. 1G, H. and Additional file 1: Fig. S1C). Additionally, the number of Tregs after treatment depended on the efficacy of the therapy. Treg levels were significantly reduced during therapy when patients achieved complete remission but remained constant in those with no remission (Fig. 1H). The effector Tregs are highly immunosuppressive cells. Furthermore, we confirmed that the proportion of CD45RA^−^ effector Tregs was significantly higher in the DLBCL group than in the control group (P < 0.0001; Fig. 1I).

### Abundance of Treg cells is increased in B lymphoma-bearing animals

**Fig. 2:**
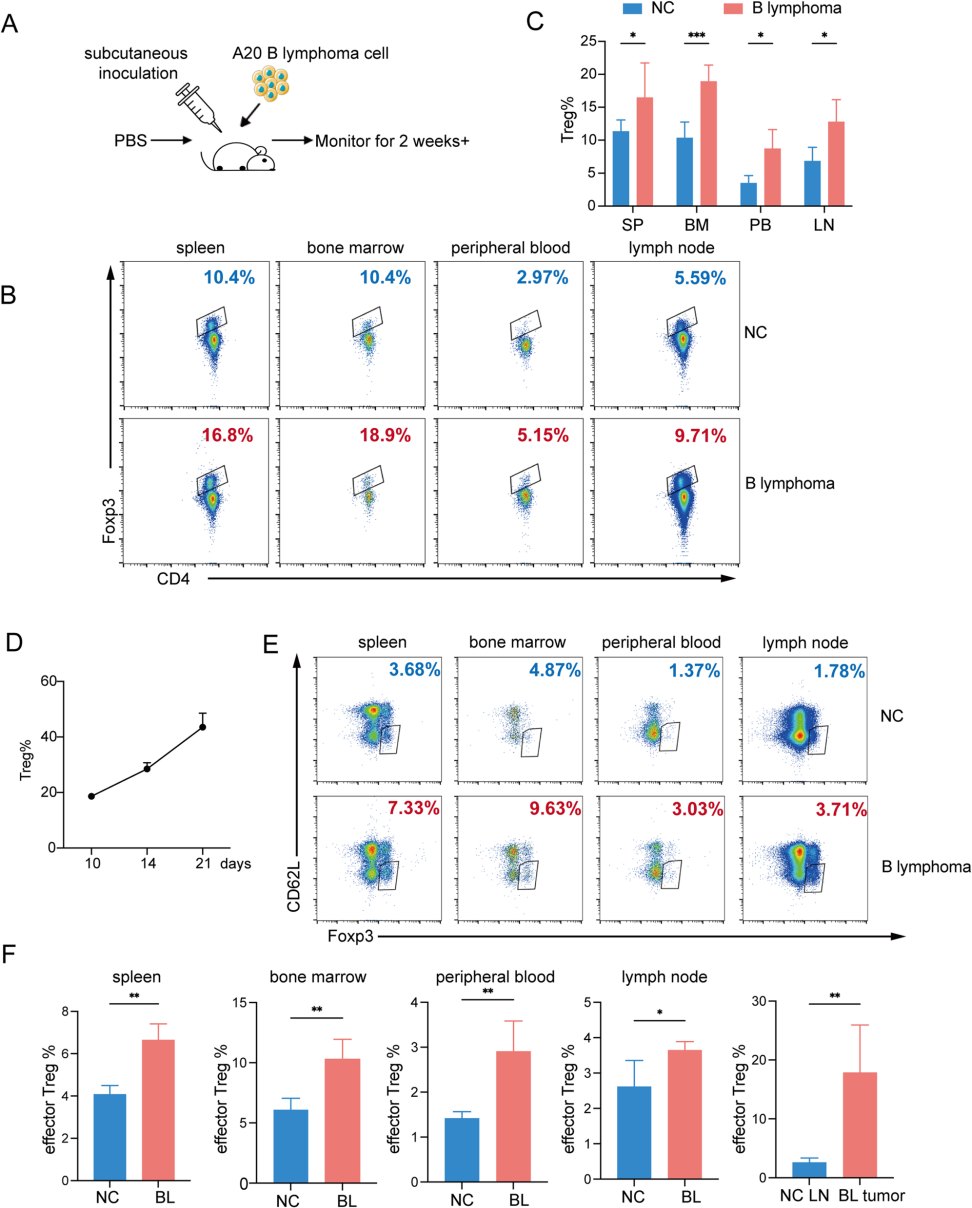
Abundance of Treg cells is increased in B lymphoma-bearing animals. **A** Schematic diagram showing the establishment of a murine B lymphoma model. Six-week-old female BALB/c mice were subcutaneously inoculated with A20 cells (5 × 10^6^ cells in 150 μL of PBS per mouse, n = 5) or PBS (n = 5) as indicated. The mice were killed and tumor and immune organs were collected. **B**, **C** Foxp3^+^Treg percentages among CD4^+^T cells in spleen (SP), peripheral blood (PB), lymph node (LN), and bone marrow (BM) from A20 B lymphoma group (BL) and control group (NC). **D** Tumor growth curves to conform the increasing proportion of Tregs over time while the tumor progressed. n = 3. **E** Representative flow cytometric analysis of CD62L^low^ effector Tregs frequencies in spleen, peripheral blood, lymph node, and bone marrow from A20 B lymphoma group (BL) and control group (NC). **F** effector Treg percentages among CD4^+^T cells in spleen, peripheral blood, lymph node, bone marrow and tumor tissue from A20 B lymphoma group and control group. *P < 0.05; **P < 0.01, ***P < 0.001. In **C**, p values were determined by two-way ANOVA; In **F**, p values were determined by two-sided unpaired t-test; data are presented as mean values ± SEM

To study the alteration in Tregs in lymphoma progression, we selected a B cell lymphoma model in which mice were subcutaneously injected with syngeneic A20 lymphoma cells (Fig. 2A). The percentage of Tregs in the spleen, bone marrow, peripheral blood, and lymph nodes from B lymphoma mice was increased compared with that in normal control mice (Fig. 2B, C and Additional file 1: Fig. S1D). Meanwhile, we found that the tumor volumes gradually increased over time, which had significantly higher percentages of Tregs in the tumor compared with the early tumor mice. We have established tumour growth curves to conform the increasing of Tregs over time while the tumour progressed (Fig. 2D), which demonstrated the correlation between Tregs and tumor progression. Additionally, there was an increase in the percentage of CD62L^low^effector Treg cells in the spleen, bone marrow, peripheral blood, lymph nodes, and tumor tissue, indicative of an immunosuppressive state in the TME (Fig. 2E-F). Taken together, these results suggest that mice with B lymphoma exhibited an immunosuppressive state characterized by increased frequencies of Tregs, especially effector Treg cells.

### Enhanced inhibitory function of Tregs from DLBCL patients

**Fig. 3:**
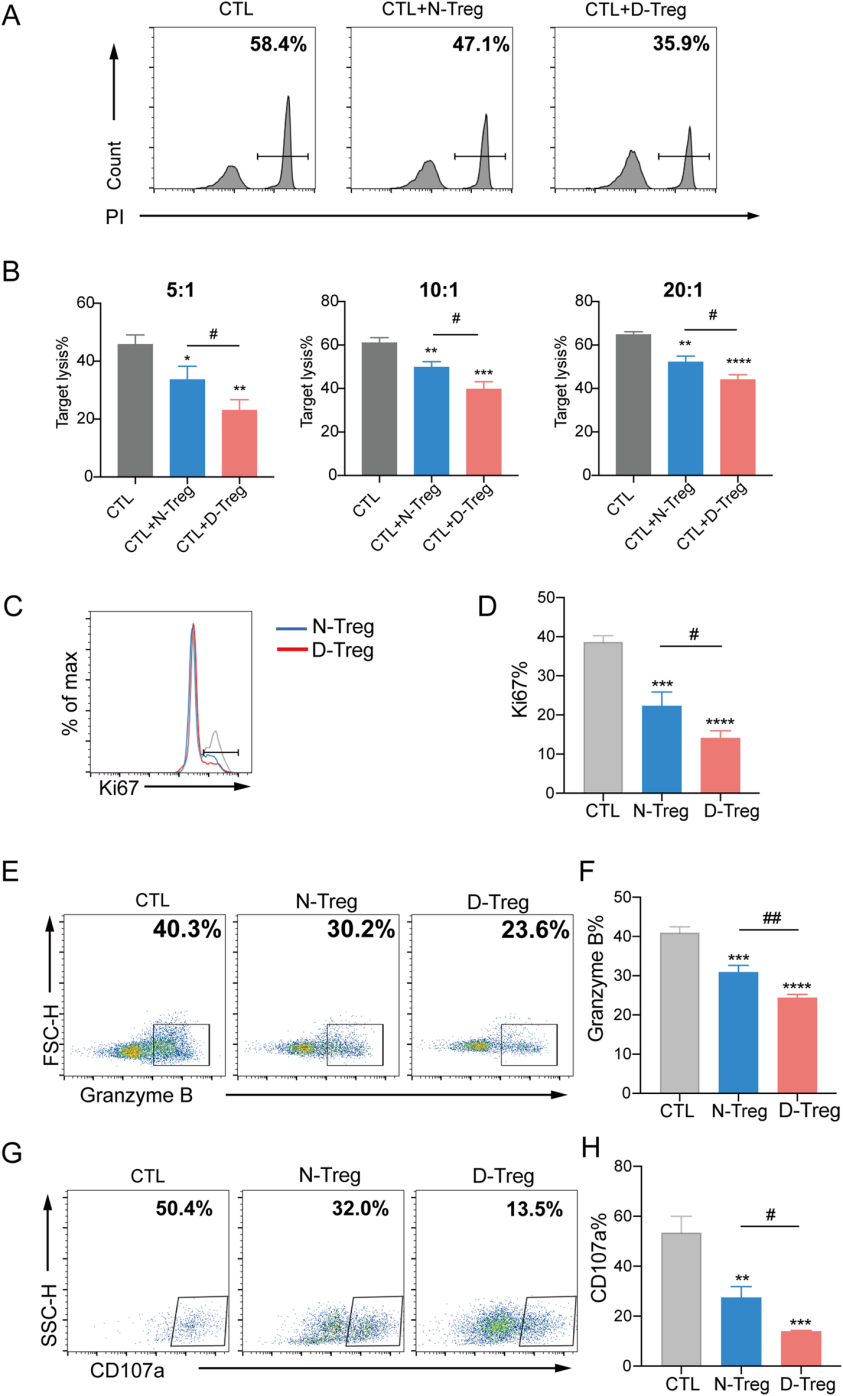
Enhanced inhibitory function of Tregs from DLBCL patients. **A** Cytotoxic activity of activated CD8^+^CTLs cocultured with or without Tregs from DLBCL patients (D-Treg) and healthy donors (N-Treg) toward CFSE-labelled SU-DHL4 cells at the E/T ratios of 10:1. The percentage of PI^+^ cells were used to calculate the % lysis of target cells by CD8^+^ CTLs. n = 3. **B** PI staining of SU-DHL4 cells from the co-culture system of CD8^+^CTLs with Tregs from DLBCL patients (D-Treg) and healthy donors (N-Treg), or without Tregs at the E/T ratios of 5:1,10:1 and 20:1. n = 3. **C**, **D** Proliferation of CD8^+^ CTLs was determined by coculturing CD8^+^ CTLs with or without Tregs from DLBCL patients (D-Treg) and healthy donors (N-Treg) for 3 days. The percentage of Ki67^+^ cells was used to calculate proliferation of CD8^+^ CTLs. n = 3. **E**, **F** Intracellular expression of GzmB in CD8^+^ CTLs cocultured either alone or with Tregs from DLBCL patients (D-Treg) and healthy donors (N-Treg) for 3 days. n = 3. **G**, **H** CD107a surface expression on CD8^+^ CTLs exposed to SU-DHL4 cells alone or in the presence of Treg cells from DLBCL patients (D-Treg) and healthy donors (N-Treg) for 4-5 h. n = 3. *P < 0.05; **P < 0.01, ***P < 0.001. Asterisk indicates significant difference from control group as assessed. Pound sign indicates significant difference from different Treg group. p values were determined by one-way ANOVA; data are presented as mean values ± SEM

One of the main functions of Tregs is to suppress effector T cells and maintain Foxp3 expression [32]. Hence, we hypothesized that the increased abundance of Tregs would contribute to immune suppression in DLBCL. To test this hypothesis, we defined the inhibitory function of Tregs on the cytotoxicity of CD8^+^ CTLs when exposed to lymphoma B cells. Using human SU-DHL4 cells as DLBCL tumor cell targets, we co-cultured activated CD8^+^ CTLs with or without PBMC-derived Tregs from patients and healthy donors at effector: target ratios of 5:1,10:1and 20:1. Tregs from DLBCL patients had stronger suppression effects on the cytotoxic activity of SU-DHL4 cells than CD8^+^CTLs alone or with Tregs from the control group (Fig. 3A, B). Next, we investigated the influence of Tregs on the proliferation of CD8^+^CTLs. After co-culture with CD8^+^ CTLs, Tregs from DLBCL patients exhibited an enhanced suppression effect on the proliferation of CD8^+^ CTLs compared with those from healthy donors (P < 0.05) (Fig. 3C, D).

The synthesis of GzmB by CD8^+^ CTLs is critical for their function [33]. GzmB is a specific cytotoxic marker for CTLs and localized in cytoplasmic granules of CTLs [34]. GzmB is released at the immune synapse by degranulation upon CTLs interaction with antigen-presenting target cells and then enters target cells and induces their apoptosis [35]. Therefore, we aimed to determine the effect of Tregs on GzmB production by activated CD8^+^ CTLs. For this purpose, we co-cultured CD8^+^ CTLs with or without Tregs from DLBCL patients or from the control group. As shown in Fig. 3E and F, we observed a stronger attenuation of the expression of GzmB in the DLBCL group compared to that in the control group. Degranulation is an important mechanism, and granule release is accompanied by the mobilization of CD107a on cytolytic cells. Therefore, CD107a surface expression is used as a marker of degranulation in cytolytic cells [36]. Hence, we assessed the effect of Tregs on CD107a expression in CD8^+^CTLs exposed to tumor cells. When activated CD8^+^ CTLs were exposed to SU-DHL4 cells with or without Tregs, CD107a surface expression was significantly reduced in the DLBCL group compared to that in the control group (P < 0.01) (Fig. 3G, H).

### Transcriptional profiling of Tregs among patients with DLBCL

**Fig. 4:**
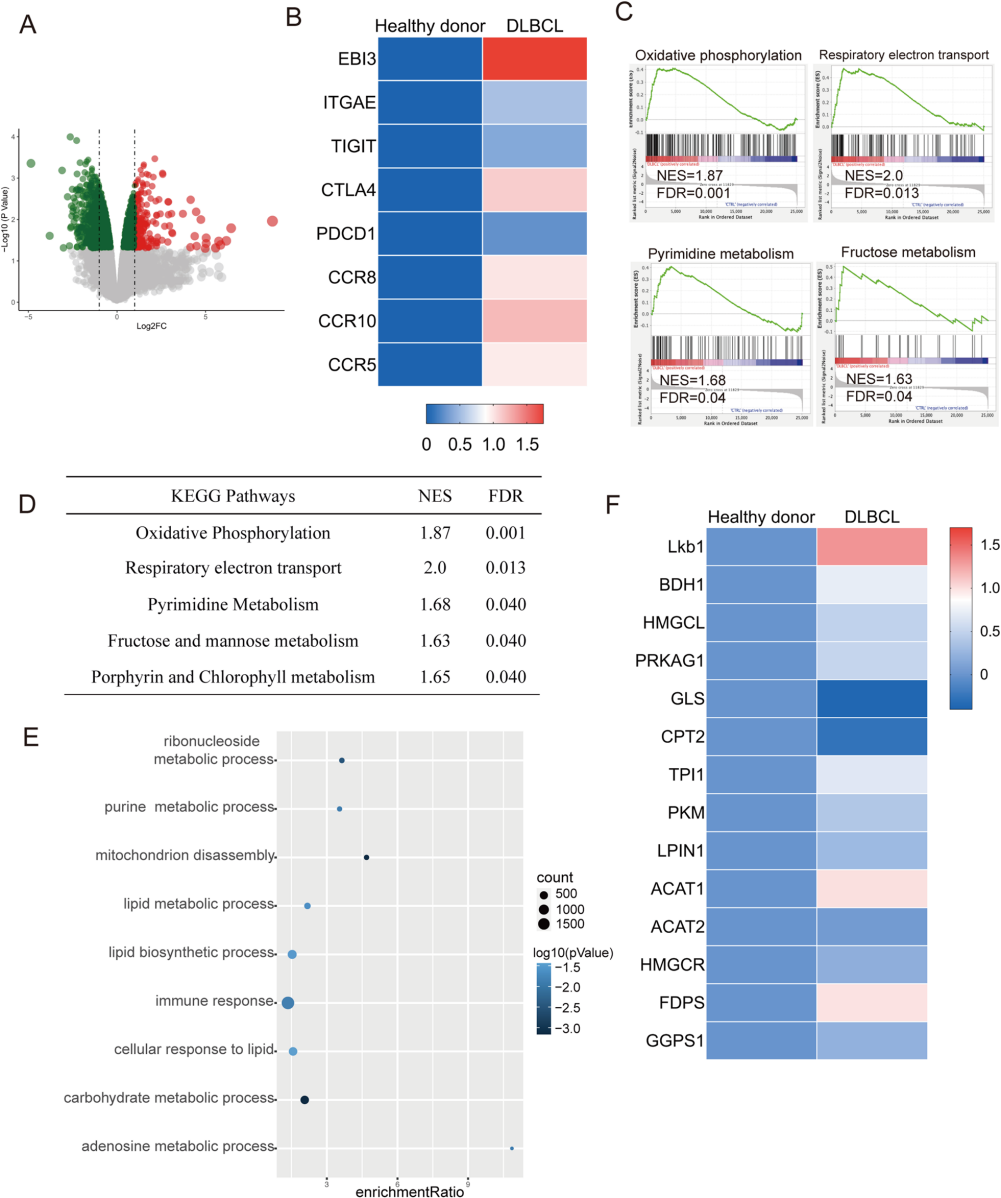
Transcriptional profiling of Tregs among patients with DLBCL. **A** DEGs in DLBCL patients compared with the healthy donors in volcano plot. **B** Tregs function-related genes differentially expressed between Tregs from DLBCL patients and healthy donors determined by transcriptional profiling. Fold difference means fold change of gene expression levels in Tregs from DLBCL patients compared with that from control group. **C**, **D** Gene pathways that were differentially expressed in Tregs from DLBCL patients versus healthy donors by Gene Set Enrichment analysis and KEGG enrichment analysis. Gene sets were considered statistically significant at an FDR P-value < 0.05. **E** GO enrichment analysis of the results using the GO database. Genes were enriched in different categories in the GO terms analysis. **F** Expression profile of Lkb1 in Tregs from RNA- sequencing and expression of lipid metabolic pathway genes of Tregs from DLBCL patients compared with that from control group from RNA- sequencing

To further understand the mechanisms behind the functional alteration of Tregs in the lymphoma environment, we assessed the transcriptional profile of Tregs sorted from healthy donors and DLBCL patients. Remarkably, DLBCL led to extensive transcriptional changes in Tregs (Fig. 4A). Interestingly, the Treg signature suppressive molecules [37], including EBI3, ITGAE, TIGIT, CTLA4, and PDCD1, were upregulated in DLBCL Tregs (Fig. 4B), in agreement with their increased in vitro suppressive function demonstrated by the previous experiments. In addition, Tregs isolated from DLBCL patients showed an upregulation of molecules, such as CCR5, CCR8, and CCR10, directing cells toward DLBCL infiltrating tissues. Pathway and gene-set enrichment analysis of the transcriptome from Tregs from DLBCL patients indicated that the upregulated genes were enriched in metabolism-related pathways, including OXPHOS, pyrimidine metabolism, fructose metabolism, and respiratory electron transport (Fig. 4C-D). Additionally, functional annotation analysis indicated that many of the dysregulated genes in DLBCL Tregs fell into functional categories associated with the immune response and metabolic homeostasis, such as lipid metabolic processes (Fig. 4E). As we all known, the role of maintenance of lipid metabolism has become critical for Treg cell biology and central to Treg activation are changes in lipid metabolism that support their survival and function [38], we want to explore whether maintenance of lipid metabolism is critical for Treg cell biology in DLBCL. Hence, we examined the expression level of representative genes of lipid metabolic pathways and found that there were many activated genes of lipid metabolism. The data uncovered that ACAT1, HMGCR, FDPS, and GGPS1 mRNA levels were elevated in Tregs from DLBCL patients (Fig. 4F) compared to those in healthy controls. Meanwhile these genes were associated with mevalonate pathway, which prompted the hyperactivation of mevalonate pathway in the DLBCL group. As previously reported [39], the mevalonate pathway plays an important role in regulating the function and phenotype of Tregs. Thus, our results suggest that Tregs in a lymphoma environment undergo considerable transcriptional rewiring of metabolic pathways.

### Lkb1 is upregulated in Tregs from DLBCL patients

Lkb1 has been demonstrated to play an important role in regulating the suppressive function of Tregs [20]. Metabolic pathways altered between the patients and donors were similar to those from microarrays in Foxp3^Cre^Lkb1^f/f^ mice [21, 22]. According to the transcriptional profiling analysis, the mRNA level of Lkb1 was much higher in Tregs from DLBCL patients than in control counterparts (Fig. 4F). Thus, it is possible that the enhanced function of Tregs in DLBCL patients might be related to the increased expression of Lkb1.

**Fig. 5:**
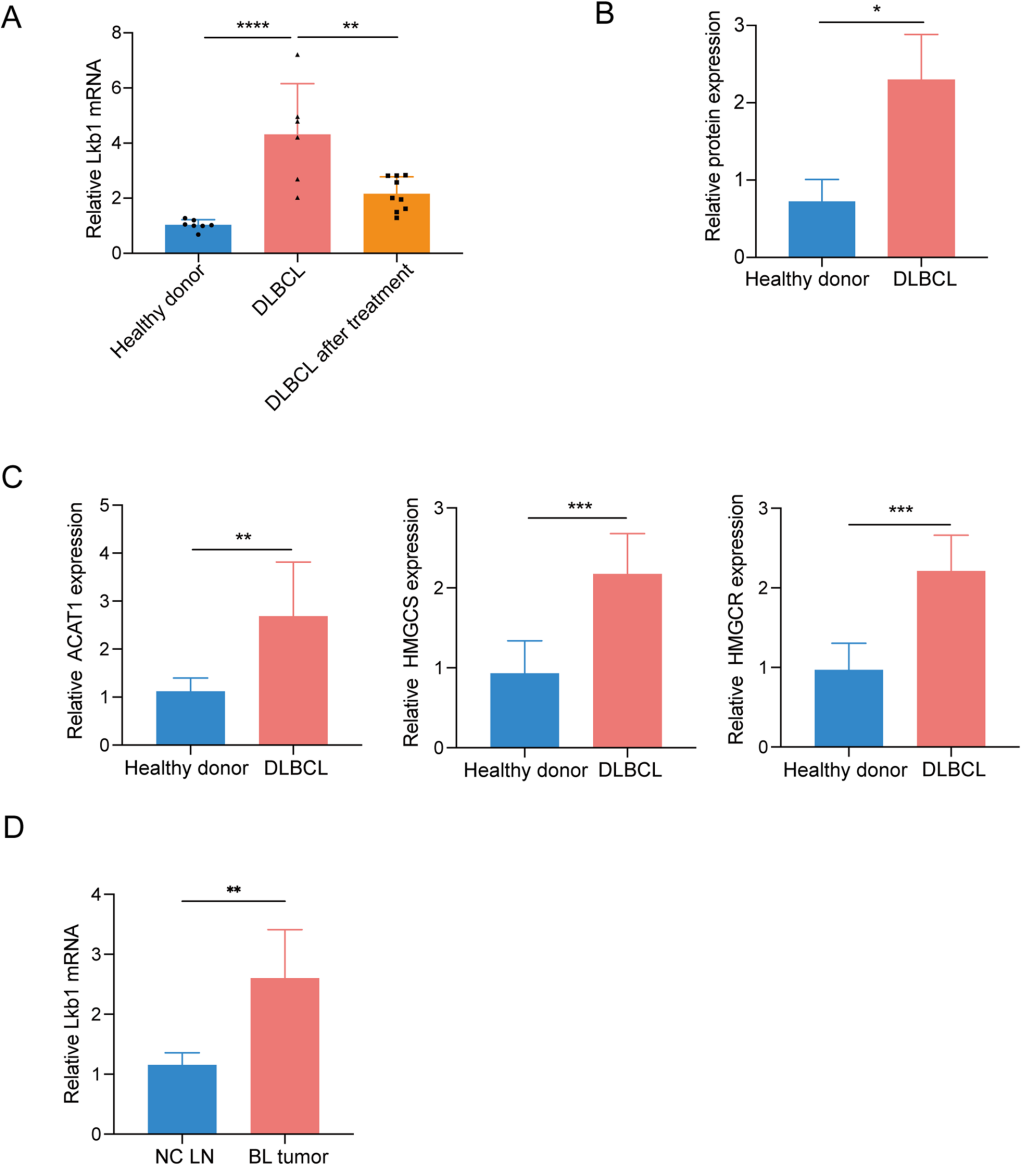
Lkb1 is upregulated in Tregs from DLBCL patients. **A** The results of qPCR were presented as mean values of relative Lkb1 mRNA expression levels to the housekeeping genes GAPDH, in healthy donors (n = 7), DLBCL patients (n = 6) and the patients after treatment (n = 9) respectively. **B** Automatic Simple Western system analysis. The ratio of the peak area of Lkb1 normalized to GAPDH from the same sample was presented, demonstrating that DLBCL patients (n = 3) had higher Lkb1 level than healthy donors (n = 3). **C** mRNA expression of mevalonate pathway genes of Tregs from DLBCL patients (n = 5) compared with that from control group (n = 7). **D** Relative Lkb1 mRNA expression levels to the housekeeping genes GAPDH, in B lymphoma and control group, respectively. n = 5. *P < 0.05; **P < 0.01, ***P < 0.001. In **A**, p values were determined by one-way ANOVA; In **B**–**D**, p values were determined by two-sided unpaired t-test; data are presented as mean values ± SEM

To further investigate this hypothesis, we analyzed the mRNA expression of Lkb1 using RT-PCR. In line with the results of transcriptional profiling analysis, the mRNA level of Lkb1 was significantly higher in Tregs from DLBCL patients than those from healthy donors (Fig. 5A). In addition, Lkb1 levels were reduced during therapy when patients achieved complete remission (Fig. 5A). Furthermore, using the Simple Western system, we detected the Lkb1 protein expression levels, and confirmed that the protein level of Lkb1 was increased in DLBCL patients compared with that in healthy donors (Fig. 5B and Additional file 3: Fig. S3A). Similarly, DLBCL patient-derived Tregs exhibited elevated ACAT1, HMGCS, and HMGCR mRNA levels compared with those derived from healthy donors (Fig. 5C), which verified the dysregulation of the mevalonate pathway. Moreover, significantly increased Lkb1 mRNA and protein levels of Tregs were observed in the tumors of B lymphoma model mice compared with those in the control group (Fig. 5D and Additional file 3: Fig. S3B). Taken together, the increasing proportion of Tregs in DLBCL might be relevant to the increased expression of Lkb1 in Tregs, and we therefore considered whether that Lkb1 plays an important role in correlation with Treg cells in DLBCL.

### Regulation of Lkb1 influences Treg function by altering the mevalonate pathway in Tregs

**Fig. 6:**
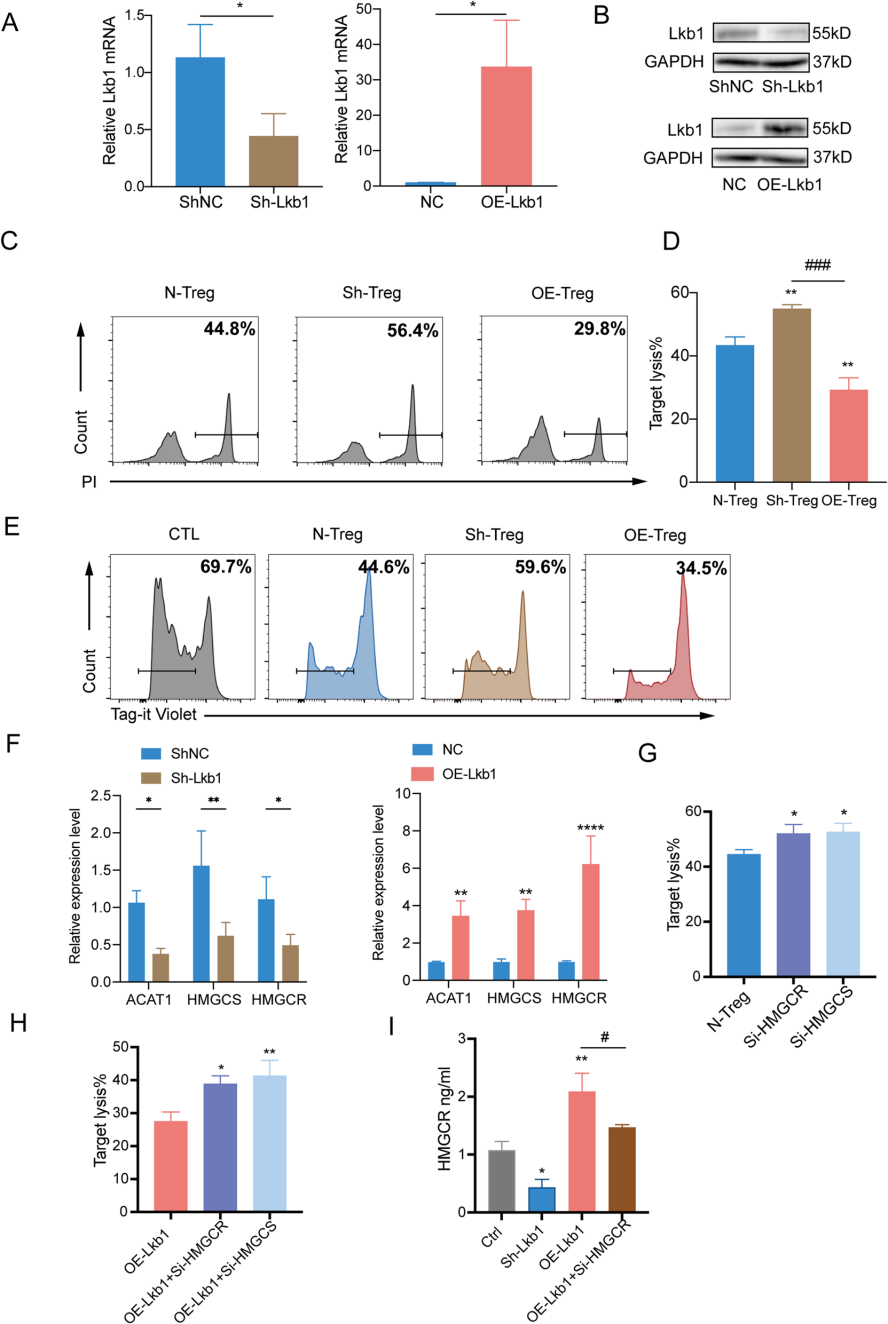
Regulation of Lkb1 influences Treg function by altering the mevalonate pathway in Tregs. **A**, **B** Determination of gene expression levels in Tregs treated by shRNA or transduced with lentivirus carrying Lkb1 complementary DNA at 48 h by qPCR analysis and western blots with indicated antibodies. n = 3. **C**, **D** Cytotoxic activity of activated CD8^+^CTLs cocultured with Tregs from healty donors (N-Treg) or Tregs with Lkb1 knock down (Sh-Treg) or overexpression (OE-Treg) toward CellTraceTM Far Red-labelled SU-DHL4 cells at the indicated E/T ratios of 10:1. The percentage of PI^+^ cells was used to calculate the % lysis of target cells by CD8^+^ CTLs. n = 3. **E** Proliferation of CD8^+^ CTLs was determined by coculturing Tag-it Violet labeled CD8^+^ CTLs with Tregs for 3 days. **F** mRNA expression of mevalonate pathway genes of Tregs with Lkb1 knock down or overexpression. n = 3. **G** PI staining of SU-DHL4 cells from the co-culture system of CD8^+^CTLs with Tregs from healty donors or with HMGCR or HMGCS interfered. n = 3. **H** PI staining of SU-DHL4 cells from the co-culture system of CD8^+^CTLs with Lkb1 overexpressed and HMGCR or HMGCS interfered Lkb1 overexpressed Treg cells. n = 3. **I** The quantity of HMGCR enzyme of Tregs were evaluated by Elisa with Lkb1 knock down or overexpression, as well as HMGCR interfered Lkb1 overexpressed Tregs. n = 3. *P < 0.05; **P < 0.01, ***P < 0.001. Asterisk indicates significant difference from control group as assessed. Pound sign indicates significant difference from different group. In A, p values were determined by two-sided unpaired t-test; In D and G-I, p values were determined by one-way ANOVA; In **F**, p values were determined by two-way ANOVA; data are presented as mean values ± SEM

Considering that the role of Lkb1 is crucial for the dysregulations of Tregs affecting tumor immune homeostasis, we aimed to verify whether the suppression effect of human Tregs was directly regulated by Lkb1. For this purpose, we silenced or overexpressed Lkb1 in Treg cells, and assessed the efficiency using RT-qPCR and western blots (Fig. 6A, B). We also perform proliferation and viability assay to characterize how the knockdown and overexpression of Lkb1 is affecting Tregs. The data showed that the survival and proliferation of Lkb1-deficient or overexpression Treg cells were barely altered compared with the control group (Additional file 4: Fig. S4A-B). The data was consistent with previous studies [20, 40] that Lkb1 mainly maintains Treg cell lineage identity by stabilizing Foxp3 expression and enforcing suppressor function rather than regulating the survival and proliferation of Tregs. Using SU-DHL4 cells as a target, we co-cultured CD8^+^ CTLs with Tregs in which Lkb1 had been silenced or overexpressed. Overexpression of Lkb1 in Tregs resulted in stronger suppression of the cytotoxic activity against SU-DHL4 cells. In contrast, Tregs in which Lkb1 expression was silenced presented decreased inhibition activity compared to the that of the control group (Fig. 6C-D). We re-performed cytotoxicity assay of CD8^+^ T cells with an additional shRNA targeting Lkb1, and data uncovered that knockdown of Lkb1 in Tregs resulted in decreased suppression of the cytotoxic activity against SU-DHL4 cells compared to the that of the control group (Additional file 4: Fig. S4C). CD8^+^ CTLs were then cultured either alone or with Tregs with in which Lkb1 was silenced or overexpressed. Tregs overexpressing Lkb1 strongly suppressed the proliferation of CD8^+^ CTLs compared to the other groups (Fig. 6E).

Lkb1 has been shown to regulate lipid metabolism, which plays important roles in coordinating Treg proliferation and function [41]. To determine the lipid metabolism in human Tregs affected by Lkb1, we examined the expression level of representative genes when Lkb1 expression was silenced or upregulated. The mRNA expression levels of ACAT1, HMGCS, and HMGCR were decreased in Lkb1-knockdown Tregs and increased in Lkb1-overexpression Tregs (Fig. 6F). The expression of HMGCR and HMGCS are known to be essential for the mevalonate pathway which is an important metabolic pathway, and HMGCR is a rate-limiting enzyme of the mevalonate pathway [42]. The protein level of HMGCR and HMGCS were also decreased in Lkb1-knockdown Tregs and increased in Lkb1-overexpression Tregs (Additional file 4: Fig. S4D). In the previous studies, indeed, Treg-specific HMGCR deletion leaded to a severe autoimmune disease in mice [43]. To further validate whether the mevalonate pathway is critical for Treg biology and whether Lkb1 affects Treg function by regulating mevalonate pathways in the progression of DLBCL, we investigated the cytotoxicity assay of CD8^+^ T cells with Tregs in which HMGCR or HMGCS had been silenced, resulting in decreased suppression function of the cytotoxic activity against SU-DHL4 cells (Fig. 6G). Moreover, we investigated the effect of HMGCR or HMGCS interference on Treg function regulated by Lkb1 overexpression. The results showed that compared with Lkb1 overexpression group, HMGCR or HMGCS interference could attenuate the strong suppression of the cytotoxic activity of CD8^+^ CTLs promoted by Lkb1 overexpression (Fig. 6H). Besides, we found that the quantity of HMGCR enzyme were regulated by expression of Lkb1 (Fig. 6I). Taken together, these results suggest that Lkb1 affects Treg function by regulating mevalonate pathways in the progression of DLBCL.

### Targeting Lkb1 expression on Tregs alleviates lymphoma tumor progression

**Fig. 7:**
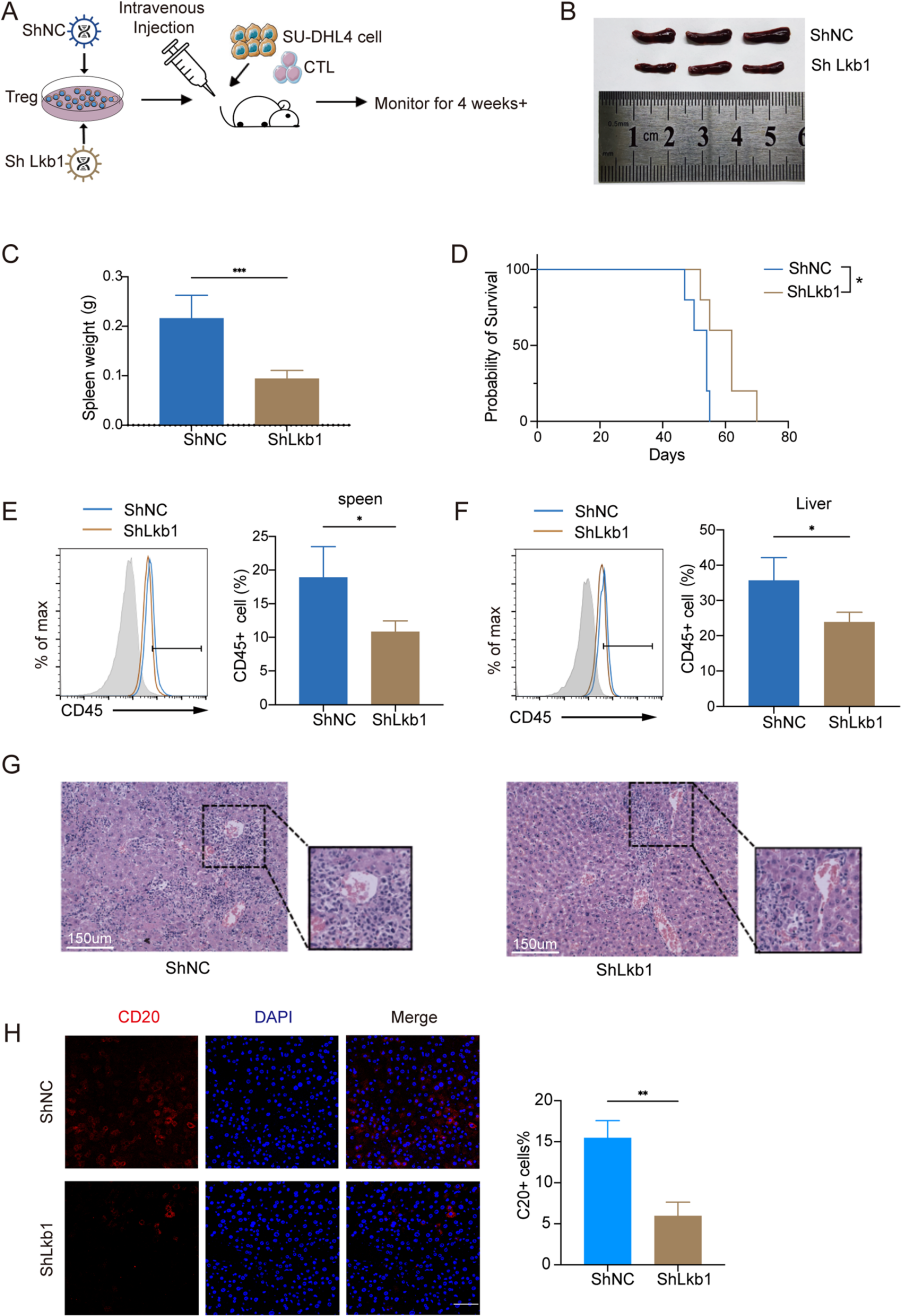
Targeting Lkb1 expression on Tregs alleviates lymphoma tumor progression. **A** Schematic design of humanized DLBCL models. Tregs was transduced with Lkb1shRNA (ShLkb1) or non-silencing scrambled control (ShNC). NSG mice were injected with Treg cells (shLkb1, n = 5) or Treg cells (ShNC, n = 5) along with SU-DHL4 cells and CD8^+^CTLs through tail vein. **B**, **C** Representative examples and summary weight data of spleens derived from two groups of lymphoma mice (n = 5). **D** For survival analysis, NSG mice were monitored until ethical euthanasia according to signs of morbidity. Survival was plotted by using the Kaplan-Meier method. **E**, **F** NSG mice from ShLkb1 and ShNC group were euthanized to evaluate tumor burden at day 35. Representative flow cytometry plots and summary data of CD45-positive cells rates in liver and spleen (n = 4). **G** Representative Hematoxylin and eosin (H&E) images from two groups showing different infiltration levels of tumor in liver. **H** Immunofluorescence staining of CD20 B cells from different groups for CD20 (red) and DAPI (blue). *P < 0.05; **P < 0.01, ***P < 0.001. p values were determined by two-sided unpaired t-test; data are presented as mean values ± SEM

Based on the previous results, we hypothesized that reducing Lkb1 expression in Tregs could alter DLBCL outcome. To test this hypothesis, we used a humanized DLBCL xenograft NSG mice model. Tregs were transduced with shRNA targeting Lkb1 (ShLkb1) or non-silencing scrambled control (ShNC) (Fig. 7A). NSG mice were engrafted with Treg cells (ShLkb1) or Treg cells (ShNC) along with SU-DHL4 cells and CD8^+^CTLs. The survival curve of mice was constructed based on the death of mice.

The lymphoma burden was evaluated by assessing the degree of organomegaly and lymphoma cell infiltration. Compared to that in the control group, spleen enlargement was lower in shLkb1 lymphoma mice, which exhibited moderate capabilities to suppress tumor growth (Fig. 7B, C). Although we only observed a relatively weak reduction in spleen infiltration in shLkb1 lymphoma mice than in control mice, adoptive transfer of Tregs in which Lkb1 expression was silenced did confer the recipient mice a pronounced survival benefit (Fig. 7D). CD45^+^ cell sorting analysis of the spleen and liver from recipient mice showed reduced proportion of lymphoma cells in shLkb1 lymphoma mice compared to that in the control group (Fig. 7E, F). Histological analysis revealed decreased pathological tumor infiltration in the livers of these animals (Fig. 7G). Consistent with the decreased infiltration in shLkb1 lymphoma mice, we also observed decreased expression of CD20 in the livers of shLkb1 lymphoma mice compared to that in the control group by immunofluorescence staining (Fig. 7H). Thus, these data indicate that targeting Lkb1 to weaken Treg function reduces the progression of lymphoma. Overall, these findings indicate that targeting Lkb1 expression on Treg cells is a promising therapeutic strategy for lymphoma.

## Discussion

In this study, we demonstrated that Tregs from DLBCL patients were more abundant than those from healthy donors and that they presented an enhanced phenotype characterized by increased suppressor function than those of healthy donors. Transcriptional analysis identified that Lkb1 in Tregs of DLBCL patients, which had been previously identified as a critical regulator of murine Treg identity, was upregulated. Additionally, silencing or overexpressing Lkb1 could affect the immunosuppressive function of human Tregs. Further experiments on DLBCL murine models verified that Lkb1 deficiency in Tregs suppresses tumor growth and promotes anti-tumor immunity.

The TME plays a vital role in various processes of B-cell lymphoma, and targeting TME components is expected to be a novel treatment strategy for B-cell lymphoma [44]. Tregs play important roles in tumor immune evasion mechanisms. In this study, we demonstrated an elevated proportion of Tregs in PBMC and in lymphatic tissues from patients with DLBCL compared to the control group. This result is consistent with results from previous studies that have reported that an increased proportion of Tregs in PBMC is a poor prognostic factor in DLBCL patients [15, 45]. However, low concentrations of Tregs in PBMC have also been observed in DLBCL patients, generating some controversy that still needs to be clarified [46, 47]. In our study, we also evaluated the effect of treatment on Treg levels. The immunosuppression caused by the accumulation of Tregs in PBMC was reduced in patients with complete remission compared with that of patients in the no remission group after therapy, reaching values presented in healthy donors. Similarly, Jiménez-Cortegana et al. reported that R2-GDP therapy induced a decrease in circulating Tregs in patients with overall survival time longer than 24 months [16]. These results further highlight the importance of studying the cell populations in the PBMC for monitoring clinical response.

Previous studies have demonstrated that Tregs can be classified into three types, and effector Tregs are highly immunosuppressive and mainly suppress anti-tumor immunity. In the present study, we found that effector Treg levels were significantly increased in DLBCL patients compared to those in healthy donors. These results suggest that targeting effector Tregs inducing their depletion or functional alterations may be a novel immunotherapy strategy. Using a lymphoma mouse model that could recapitulate many characteristics of human DLBCL [48], we confirmed that this may be a valid approach.

The above results raise the question of how increasing Tregs emphatically regulates Treg function, contributing to immune surveillance against DLBCL. Similar to intratumoral Tregs isolated from tissue biopsy specimens from B-cell non-Hodgkin lymphoma patients [49], in our study, Tregs from DLBCL patients displayed an enhanced ability to suppress CD8^+^ CTLs. In addition, since blocking the migration of Tregs to the TME is a promising strategy for reducing the infiltration of Tregs, we hypothesized that these cells could be recruited from the circulation. Previous reports indicated that chemokine gradients, such as CCR5/CCL5, CCR8/CCL1, and CCR10/CCL28, can be involved in recruiting Tregs into the TME [50–52]. Interestingly, our transcriptome analysis revealed that the genes encoding the chemokine receptors CCR5, CCR8, and CCR10 were significantly upregulated in DLBCL compared to those in the normal tissues. Additionally, the expression levels of these molecules were correlated with their migration capacity to the tumor tissues in lymphoma. Hence, our data suggest a systemic immune suppression state and dysregulation of the tumor immune microenvironment in DLBCL.

Lkb1 has been shown to be an important factor for the proliferation of thymocytes and peripheral T-cells [53]. Previous research has verified that Lkb1 may be related to the stability of Tregs [20]. The regulation of Lkb1 in Tregs also contributes to the activation of β-catenin signaling [54]. Besides, the function of Lkb1 in Tregs has been reported to be largely independent of the AMP-activated protein kinase but mediated by MAP/microtubule affinity-regulating kinases and salt-inducible kinases [21]. While these studies focused on the role of Lkb1 in autoimmune diseases, we focused on the regulatory role of Lkb1 in the tumor immune microenvironment. To the best of our knowledge, ours is the first study to report that Lkb1 was significantly upregulated in Tregs from DLBCL patients compared with that in the control group, and that the upregulation of Lkb1 plays an important role in the regulation of Treg function and the immune environment of DLBCL.

Tregs exhibit a unique metabolic profile in the low-glucose, high-extracellular lactate TME [55]. Our results showed a close correlation between immune response and maintenance of metabolic homeostasis, including lipid metabolic processes and the activation of OXPHOS in DLBCL Tregs. Therefore, determining the metabolic pathway in Tregs affected by Lkb1 would greatly facilitate the development of targeted therapies. To evaluate the regulatory mechanisms in DLBCL patients, we further performed functional in vitro experiments. Our results suggest that the Lkb1-activated mevalonate pathway may regulate certain aspects of Treg function in the DLBCL environment.

Tregs suppress anti-tumor responses. The ablation of Tregs, which permits effector cells to exert a cytotoxic function, has been shown to significantly delay tumor development and to be an effective treatment for some cancers [56]. In the present study, we interpreted the role of Lkb1 expressed on Tregs in the downregulation of tumor immunity. Our data suggested that the regulation of the mevalonate pathway by Lkb1 in Tregs heavily influences lipid metabolism and is a critical mechanism for anti-tumor immunity, suggesting that targeting it may be a novel potential therapy strategy for lymphoma. Nonetheless, anti-tumor therapy may cause immunotherapy-related adverse effects, limiting its clinical benefits [57]. Therefore, more studies should be conducted to find a therapy in which a balance between anti-tumor immunity and autoimmunity is achieved.

In conclusion, Lkb1-mediated mevalonate pathway activation in lymphoma was responsible for the increased abundance and suppressive activity of Tregs in DLBCL. Our results highlight the importance of effector Tregs in the TME and in DLBCL and the importance of studying their regulatory mechanisms to further understand tumor occurrence. Additionally, our results provide novel insights into the design of immunotherapeutic strategies for DLBCL and support the genetic reprogramming of Tregs to inhibit Lkb1 or Lkb1-related pathways as an attractive strategy for anti-tumor immunotherapy. In fact, exploring how Lkb1 expression is up-regulated in Tregs during the disease process of DLBCL is very important for targeting Lkb1- related pathways for anti-tumor immunotherapy of DLBCL. Although many downstream pathways mediating the function of Lkb1 have been described, little is known about the upstream mechanisms regulating Lkb1 activity. Previous studies have reported the regulation of Lkb1 at protein levels, such as SIRT1 and SIRT2 functioned as the upstream regulators for Lkb1/AMPK signaling and played essential roles in the metabolic diseases [58, 59]. We will try to explore further mechanisms about how Lkb1 expression is up-regulated in Treg cells from DLBCL and highlight Lkb1-related pathways as therapeutic targets. Additional studies are required in the future to understand the comprehensive mechanistic landscape of Tregs.

## Conclusion

Our results demonstrate that Tregs derived from the DLBCL patients present a specific phenotype, characterized by increased abundance, enhanced functions and metabolic disturbance, especially of effector Tregs. Additionally, we demonstrated that Lkb1 contributes to Treg immunosuppressive function in DLBCL by regulating the mevalonate pathway. These results revealed an important mechanism of lymphoma immune escape mediated by Tregs, and suggest that Lkb1-related pathways may be potential therapeutic targets for DLBCL.

## Supplementary Information

**Additional file 1: Fig. S1.** Enrichment gating strategy of the percentage of Foxp3^+^Treg cells in human CD3^+^CD4^+^CD25^high^ CD127^low/neg^ Treg cells. (B) Enrichment gating strategy of human CD3^+^CD4^+^CD25^high^ CD127^low/neg^ CD45RA^−^ effector Treg cells in lymphatic tissues. (C) Enrichment gating strategy of human CD3^+^CD4^+^CD25^high^ CD127^low/neg^ CD45RA^−^ effector Treg cells in PBMC. (D) Enrichment gating strategy of mice CD3^+^CD4^+^Foxp3^+^CD62L^−^ effector Treg cells in spleen.

**Additional file 2: Fig. S2.** Heatmaps showing the top (by fold change) marker genes for each subset. The fold change means the values of normalized expression of genes in a specific subset compared to the normalized expression of genes in the other subsets. (B) T cells from AML patients (n = 5) and control group (n = 4) and (D) T cells from T lymphoma patients (n = 5) and control group (n = 4) biologically independent samples were combined and visualized using t-SNE. The cells were coloured with respect to their cluster. Proportion of Treg cells and Treg subpopulations identified in samples from the two groups based on scRNA-seq in pie charts in AML (C) and T lymphoma (E) compared with healthy donors.

**Additional file 3: Fig. S3.** (A)Automatic Simple Western system analysis. The peaks at 40 kDa and 57 kDa represented GAPDH and Lkb1, respectively. The ratio of the peak area of Lkb1 normalized to GAPDH from the same sample was presented. (B) Lkb1 proteins in CD4^+^ CD25^high^ Tregs from mice spleen cells in B lymphoma and control group by Simple Western system.

**Additional file 4: Fig. S4.** (A)Proliferation of Tregs treated by shRNA or transduced with lentivirus carrying Lkb1 complementary DNA. The percentage of Ki67^+^ cells was used to calculate proliferation of Tregs. n = 3. (B) Cell apoptosis was measured by flow cytometry. The cell apoptotic rates between different groups were analyzed in Tregs. n = 3. (C) Cytotoxic activity of CD8^+^CTLs cocultured with Tregs that with Lkb1 knock down (Sh2-Treg) toward SU-DHL4 cells. The percentage of PI^+^ cells were used to calculate the % lysis of target cells by CD8^+^ CTLs. n = 3. (D) Western blot analyzed the expression of HMGCR and HMGCS in Tregs with Lkb1 knock down or overexpression. In A-B, p values were determined by one-way ANOVA; In C, p values were determined by two-sided unpaired t-test; data are presented as mean values ± SEM.

**Additional file 5: Table S1**. The list of top 50 up and down-regulated genes.

**Additional file 6: Fig. S1.2:** The sequences of the primers used for RT-PCR.

## Data Availability

The datasets used and/or analyzed during the current study are available from the corresponding author on reasonable request.

## Abbreviations

CFSE: 5,6-Carboxyfluorescein-diacetatesuccinimidyl-ester ester
CTL: Cytotoxic T cells
DLBCL: Diffuse large B-cell lymphoma
FBS: Fetal bovine serum
GzmB: Granzyme B
Lkb1: Liver kinase B1
PBMC: Peripheral blood mononuclear cell
TME: Tumor microenvironment
Tregs: Regulatory T cells
