# Supplementary figures and images for: Lkb1 aggravates diffuse large B-cell lymphoma by promoting the function of Treg cells and immune escape
Source: J Transl Med. 2022 Aug 19;20:378. doi: 10.1186/s12967-022-03588-0 (PMC9392310; doi:10.1186/s12967-022-03588-0)

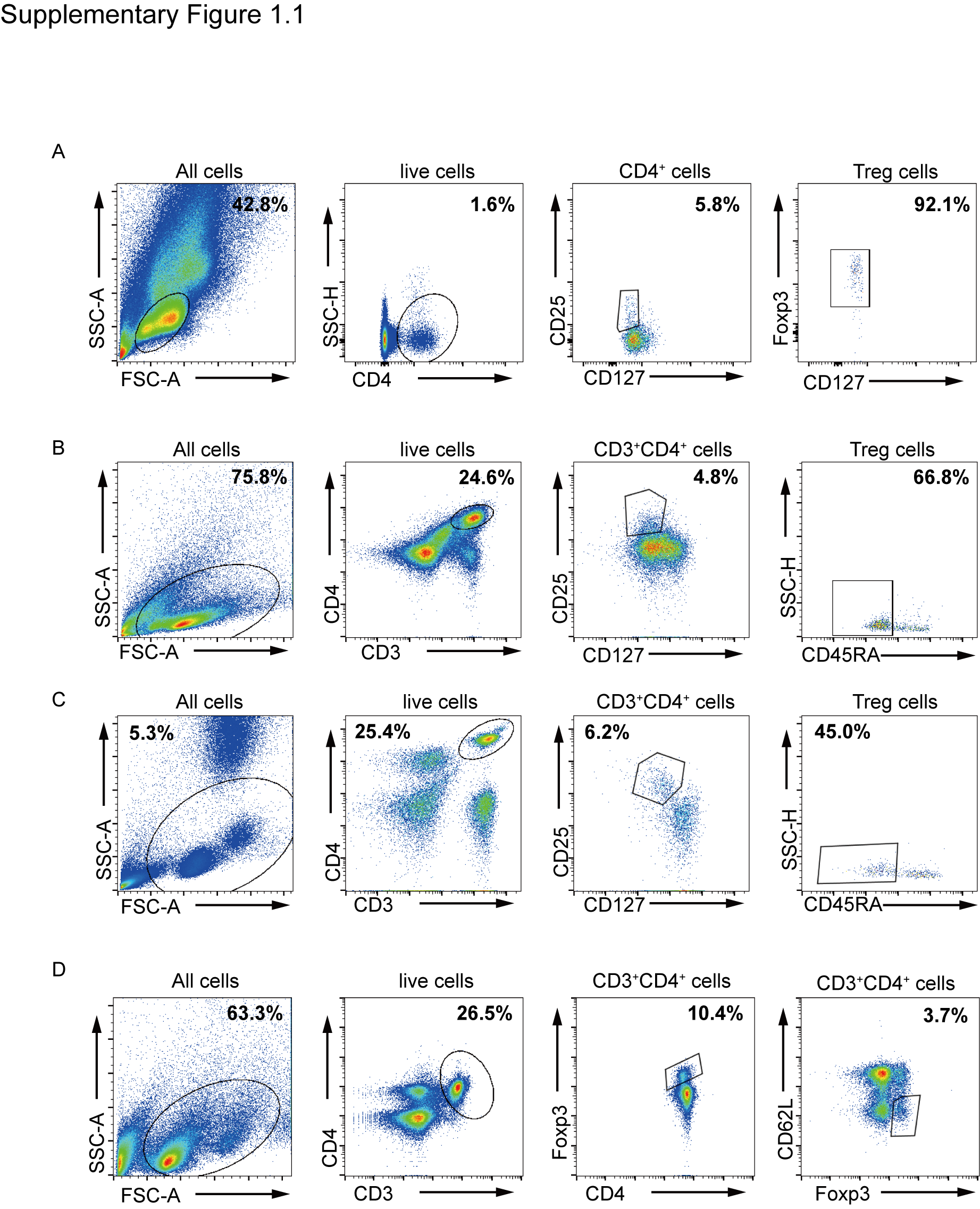

Supplement: Supplementary file 1 — Additional file 1: Fig. S1. Enrichment gating strategy of the percentage of Foxp3+Treg cells in human CD3+CD4+CD25high CD127low/neg Treg cells. (B) Enrichment gating strategy of human CD3+CD4+CD25high CD127low/neg CD45RA− effector Treg cells in lymphatic tissues. (C) Enrichment gating strategy of human CD3+CD4+CD25high CD127low/neg CD45RA− effector Treg cells in PBMC. (D) Enrichment gating strategy of mice CD3+CD4+Foxp3+CD62L− effector Treg cells in spleen. [file 12967_2022_3588_MOESM1_ESM.png]

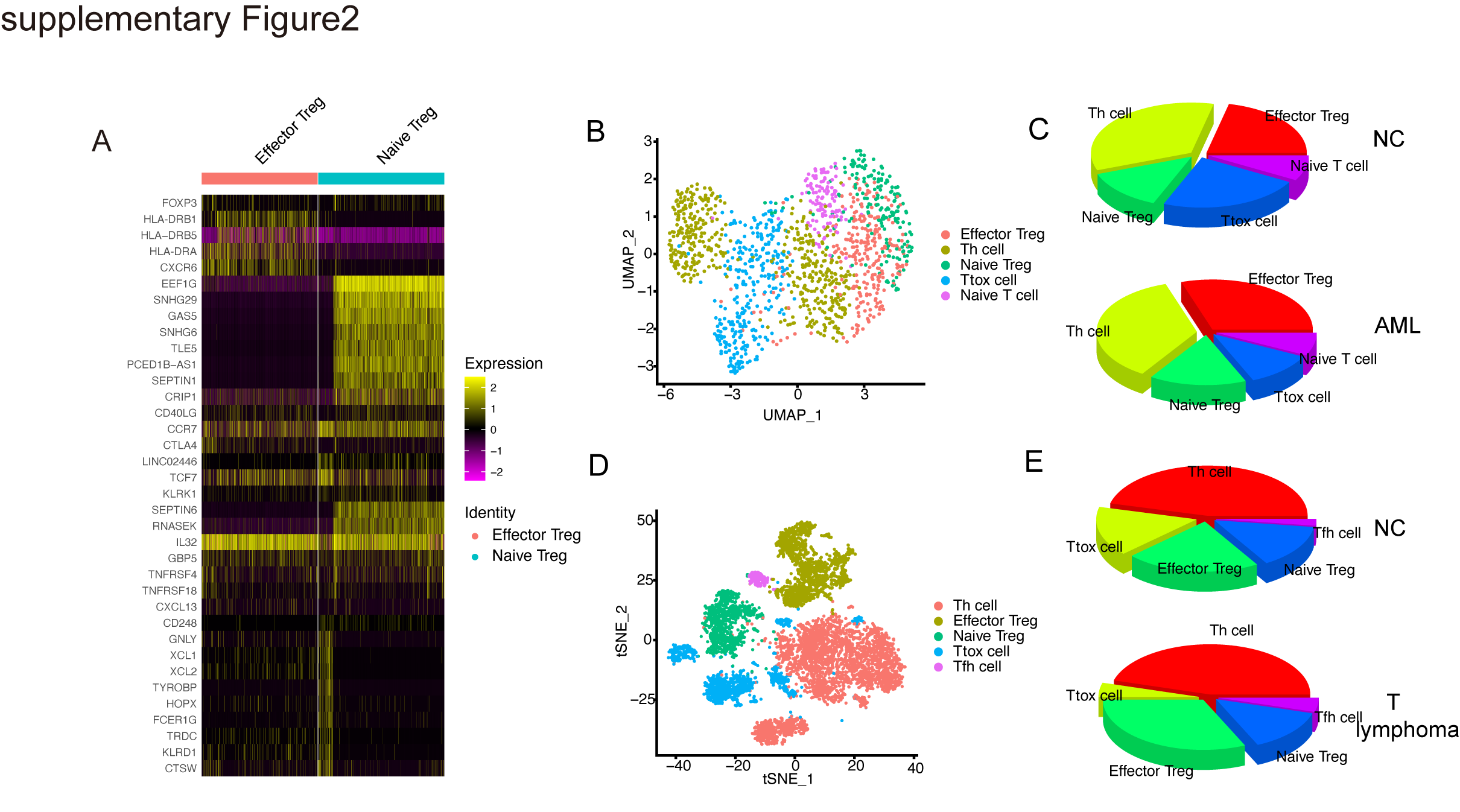

Supplement: Supplementary file 2 — Additional file 2: Fig. S2. Heatmaps showing the top (by fold change) marker genes for each subset. The fold change means the values of normalized expression of genes in a specific subset compared to the normalized expression of genes in the other subsets. (B) T cells from AML patients (n = 5) and control group (n = 4) and (D) T cells from T lymphoma patients (n = 5) and control group (n = 4) biologically independent samples were combined and visualized using t-SNE. The cells were coloured with respect to their cluster. Proportion of Treg cells and Treg subpopulations identified in samples from the two groups based on scRNA-seq in pie charts in AML (C) and T lymphoma (E) compared with healthy donors. [file 12967_2022_3588_MOESM2_ESM.png]

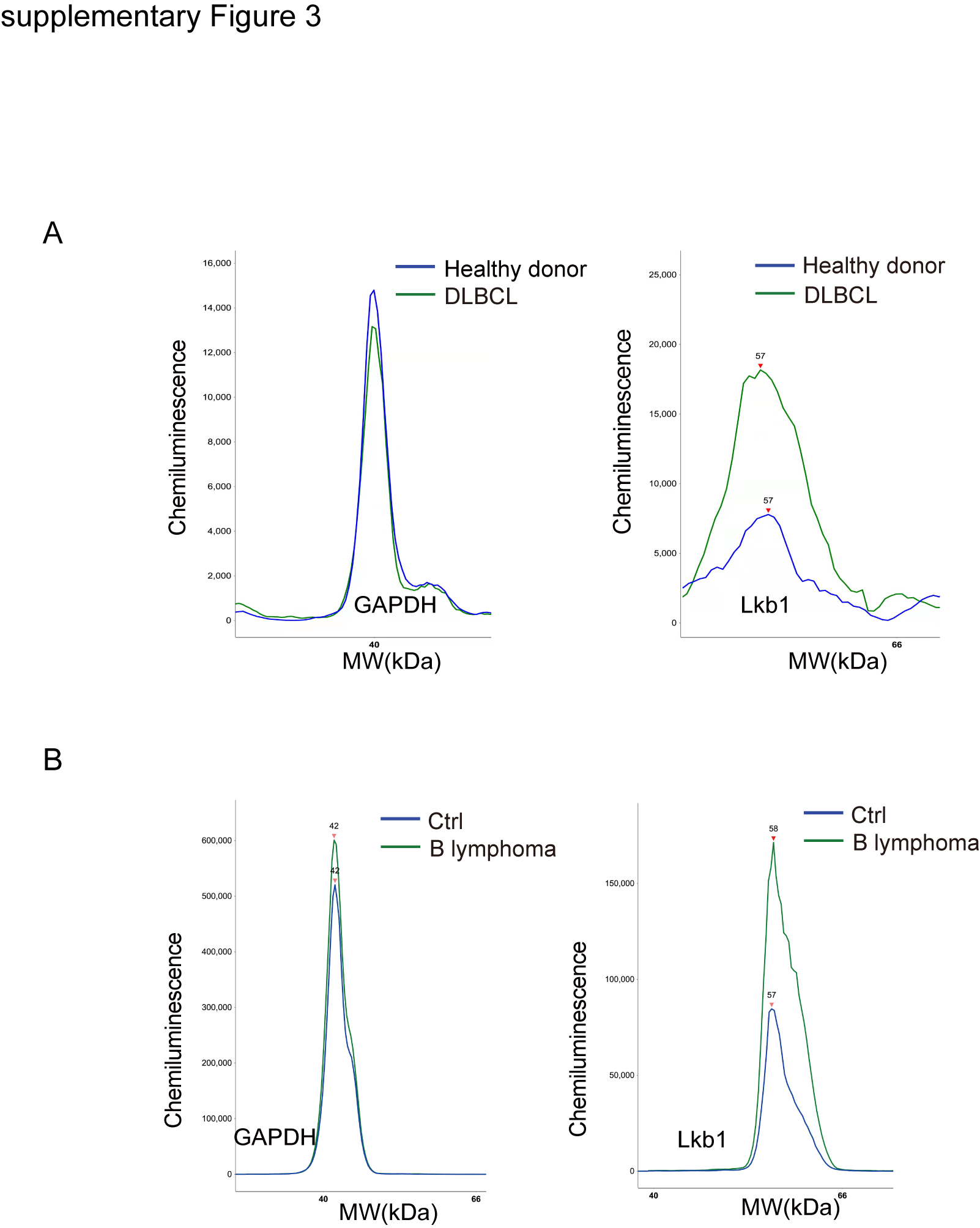

Supplement: Supplementary file 3 — Additional file 3: Fig. S3. (A)Automatic Simple Western system analysis. The peaks at 40 kDa and 57 kDa represented GAPDH and Lkb1, respectively. The ratio of the peak area of Lkb1 normalized to GAPDH from the same sample was presented. (B) Lkb1 proteins in CD4+ CD25high Tregs from mice spleen cells in B lymphoma and control group by Simple Western system. [file 12967_2022_3588_MOESM3_ESM.png]

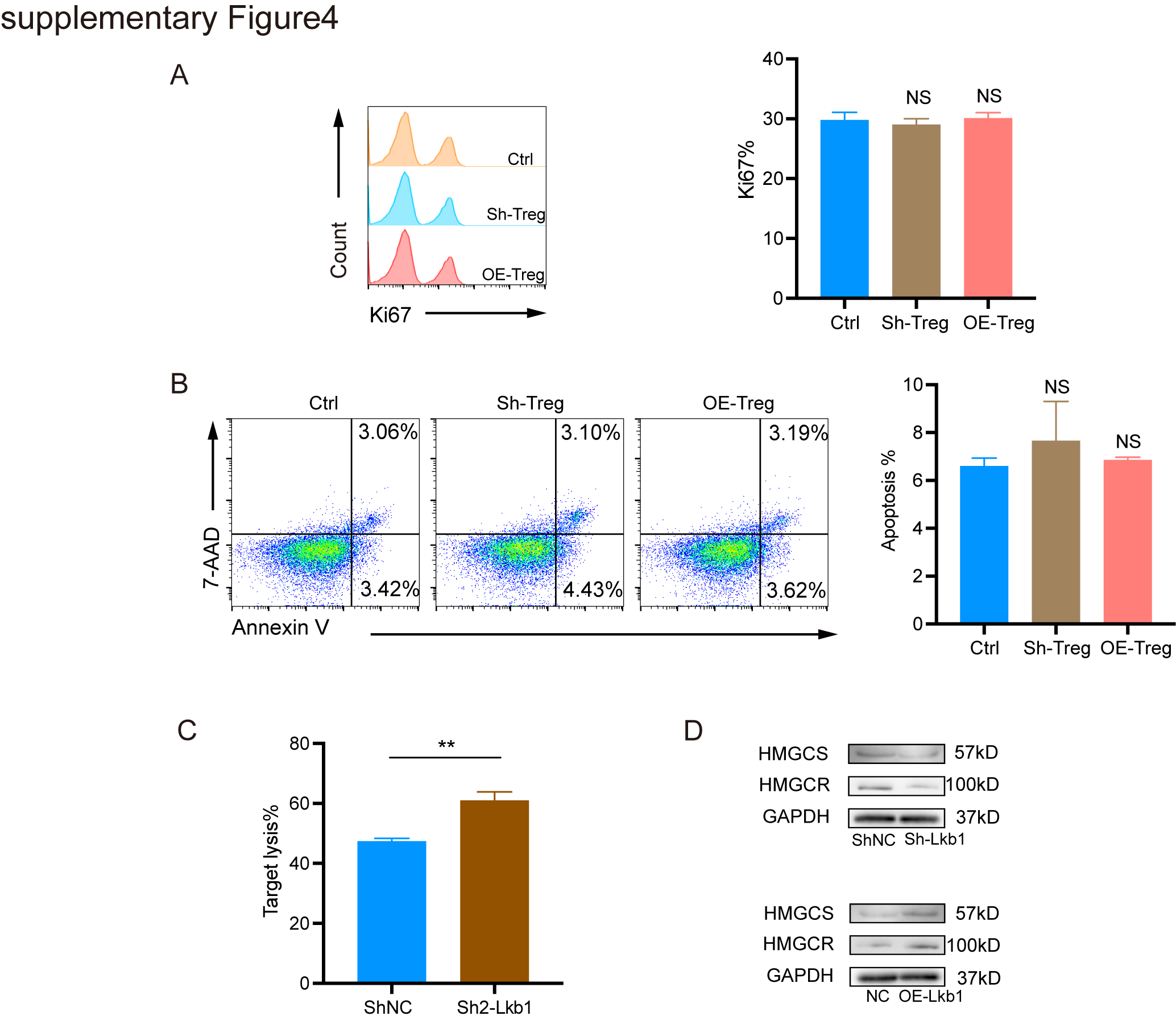

Supplement: Supplementary file 4 — Additional file 4: Fig. S4. (A)Proliferation of Tregs treated by shRNA or transduced with lentivirus carrying Lkb1 complementary DNA. The percentage of Ki67+ cells was used to calculate proliferation of Tregs. n = 3. (B) Cell apoptosis was measured by flow cytometry. The cell apoptotic rates between different groups were analyzed in Tregs. n = 3. (C) Cytotoxic activity of CD8+CTLs cocultured with Tregs that with Lkb1 knock down (Sh2-Treg) toward SU-DHL4 cells. The percentage of PI+ cells were used to calculate the % lysis of target cells by CD8+ CTLs. n = 3. (D) Western blot analyzed the expression of HMGCR and HMGCS in Tregs with Lkb1 knock down or overexpression. In A-B, p values were determined by one-way ANOVA; In C, p values were determined by two-sided unpaired t-test; data are presented as mean values ± SEM. [file 12967_2022_3588_MOESM4_ESM.png]
