# Supplementary material for: Lkb1 aggravates diffuse large B-cell lymphoma by promoting the function of Treg cells and immune escape
Source: J Transl Med. 2022 Aug 19;20:378. doi: 10.1186/s12967-022-03588-0 (PMC9392310; doi:10.1186/s12967-022-03588-0)
Supplement: Supplementary file 5 — Additional file 5: Table S1. The list of top 50 up and down-regulated genes. [file 12967_2022_3588_MOESM5_ESM.pdf]

Supplementary Table1: The list of top 50 up and down-regulated genes

Top 50 up-regulated genes	Top 50 down-regulated genes
G0S2	RNVU1-14
CCL3	SNORD68
CXCL8	AP005019
CD14	FKBP2
CCL3L1	RGPD1
FPR1	LAIR2
TYROBP	SNORD89
S100A8	LINC02482
FOS	TRBV11-3
CDA	EID2
CCL4	HIST1H4J
RETN	PABPC1P3
CXCR1	ADCK5
FTH1P10	AC244213
IL1B	MT-TN
MIR22	TRBV16
PLBD1	AC025257
LILRA5	METTL27
SENP3-EIF4A1	TRBV7-7
TRGC2	JAKMIP1
HSPA1B	MT-TA
NFIL3	MT-TC
HILPDA	ARMC6
KLF10	CD52
GSTM1	AC006077
MTCO2P12	NELFB
DUSP1	SUN2
KIR2DL3	TRBV15
CD300LF	PI16
AL049836	AC073389
MIR3945	RNVU1-7
CD83	USP18
CLEC4A	PHBP9
NFKBIA	BX293535
SGK1	SNORA28
FTH1P7	AGMAT
HIST2H2AA4	TRAV40
TMSB4XP1	SIRT1
CA2	AC147067
FTH1P8	PMVK

PRKCD	PARP10
HSPA1A	NDUFAF1
HAVCR2	ABALON
IER5	AL445472
SERTAD1	TNK1
GPR137B	AC079807
OR7E38P	STMN3
TRAPPC5	MIF-AS1
RNU6-7	NME1-NME2
FTH1P23	AC139720
