# Supplementary material for: Lkb1 aggravates diffuse large B-cell lymphoma by promoting the function of Treg cells and immune escape
Source: J Transl Med. 2022 Aug 19;20:378. doi: 10.1186/s12967-022-03588-0 (PMC9392310; doi:10.1186/s12967-022-03588-0)
Supplement: Supplementary file 6 — Additional file 6: Fig. S1.2: The sequences of the primers used for RT-PCR. [file 12967_2022_3588_MOESM6_ESM.pdf]

Supplementary material 1.2: The sequences of the primers used for RT-PCR.

Primer	5'to3'
STK11-Forward	GAGGAGGTTACGGCACAAA
STK11-Reverse	CGCAGTACTCCATCACCATATAC
GAPDH-Forward	GGTGGTCTCCTCTGACTTCAACA
GAPDH-Reverse	GTTGCTGTAGCCAAATTCGTTGT
ACAT1-Forward	AGACGGGCTAACTGATGTCT
ACAT1-Reverse	GCGTCCTGTTCATTTCGTGC
HMGCR-Forward	CATAGGAGGCTACAACGCCC
HMGCR-Reverse	ACCACCCACCGTTCCTATCT
HMGCS-Forward	TGGGAATTGTTGCCCTTGAG
HMGCS-Reverse	ACCACAGTCATGCAAAGAGAGT
